# Relationship Between Cognitive Appraisal of Control and Cardiac Vagal Regulation During an Unsupported Ski Crossing of Greenland

**DOI:** 10.3389/fphys.2022.804710

**Published:** 2022-04-08

**Authors:** Pietro Trabucchi, Aldo Savoldelli, Laurent Mourot, Philippe Vacher, Barbara Pellegrini, Federico Schena

**Affiliations:** ^1^Dipartimento Neuroscienze, Biomedicina e Movimento, Università di Verona, Verona, Italy; ^2^Centro di Ricerca “Sport, Montagna e Salute”, Università di Verona, Rovereto, Italy; ^3^Research Unit EA3920 Prognostic Markers and Regulatory Factors of Cardiovascular Diseases and Exercise Performance, Health, Innovation Platform, University of Bourgogne Franche-Comté, Besançon, France; ^4^Division for Physical Education, Tomsk Polytechnic University, Tomsk, Russia; ^5^Research Center for Education Learning and Didactics (EA 3875), University of West Brittany, Brest, France

**Keywords:** cognitive appraisal of (un)certainty, stress management, cardiac vagal regulation, unsupported expedition, ski crossing, hrv (heart rate variability), ultra-endurance, psychological resilience

## Abstract

**Purpose:**

The aim of the present study was to investigate the relationships between Perceived Control (PC) and Heart rate variability (HRV) during a 27-day expedition, during which an unsupported crossing was made from the west coast to the east coast of Greenland (across the Ice Sheet); and that therefore a high PC represents a favourable factor for recovery and stress management.

**Methods:**

Four subjects participated in the study. PC was measured on alternated days in the evening at the end of the day, using the Pearlin Mastery Scale; and the next day, upon waking, heart rate using a wrist heart rate monitor and a chest strap. Together with the PC, the perceived effort was measured through the CR-100 Borg scale and each subject was asked to indicate the most emotionally significant event of the day.

Time and frequency domain indices for heart rate variability were calculated.

**Results:**

Several correlations were observed between PC and HRV indices. In particular two indices in the time domain, standard deviation of all NN intervals (SDNN) (rrm = 0.51) and root mean square of successive (RMSSD) (rrm = 0.46), showed a significant and strong positive correlation.

**Conclusion:**

The existence of a positive correlation between PC and cardiac vagal regulation is of great interest to individuals immerged into extreme situations, because it can affect performance or prevents maladaptive states or injuries. To improve stress management, it could be convenient for members of extreme expeditions to adopt forms of cognitive training that modify their cognitive appraisal in order to raise their perception of control.

## Introduction

Perceived control (PC) can be defined as an individual’s belief about his or her own ability to exert influence on their internal states, behaviour, external environment or goal attainment. Since J. B. Rotter’s initial formulation in 1966 (Rotter, 1966; Skinner, 1996), PC has been regarded as a cognitive process capable of modifying a subject’s behaviour, physiological functioning and even his or her own survival. For example, Martin Seligman’s seminal studies (Seligman, 1972) demonstrated that systematic manipulation of PC can lead people (as well as non-human primates or mammals) to experience dysfunctional and anti-survival internal states, such as “learned helplessness.”

A large body of research indicates that PC may have protective effects on health (Karasek, 1998; Steptoe and Wardle, 2001; Lachman et al., 2011). An interesting question in this context is whether changes in PC may have an impact on physiological stress levels. Indeed, since the 1990s an increasing number of studies have confirmed a relationship between PC and physiological stress responses (Bollini et al., 2004; Gilchrist et al., 2015).

Some studies have focused in particular on the relationship between PC and heart rate variability (HRV) values. One such study showed that an increase in PC achieved through the use of cognitive techniques for coping with everyday life events was associated with an improvement in HRV (Schwerdtfeger et al., 2019).

One area where the PC of a subject may have a strong impact on his or her performance or capability of exerting influence on their external environment and goal attainment is in the context of polar expeditions. The polar environment presents explorers with many potential sources of stress and high levels of uncertainty. Physical stressors include the presence of crevasses, very strong winds and blizzards, the effect of 24 h of daylight, extreme temperatures, rugged terrain, the presence of polar bears and—in some areas of Antarctica as well as in Greenland, on the ice cap—altitudes approaching 3,000 m. Personal stressors may include interpersonal conflict within the team, fatigue, difficulty in sleeping and recovering, doubts about goal attainment or one’s performance, and negative changes in mood (Palinkas and Suedfeld, 2008).

To date, relatively few studies in this field have focussed on the description and understanding of how psycho-physiological variables are altered in ecological situations, such as long Arctic and Antarctic crossings, where subjects live in direct contact with the environment for long periods (Bishop et al., 2001). Most studies, in fact, focus on individuals staying at Antarctic bases, who are much easier to monitor (Wood et al., 2005; Sarris, 2007; Palinkas and Suedfeld, 2008).

Since the PC of those who venture into the polar environment is challenged by many stressors, it would be interesting to study their impact, in terms of physiological stress.

However, to the best of our knowledge no studies have focussed specifically on this topic. PC has only been taken into account in a study on a solo female expedition to the North Pole. The study focussed on the explorer’s daily entries in her diary and the structured interviews she underwent for research purposes. In the report, PC was correctly considered within the broader context of those factors which enhance coping resources (Devonport et al., 2011). However, in this case PC was only indirectly evaluated, nor was it correlated with physiological variables.

Hence, the aim of the present study was to investigate the relationships between PC and HRV during a 27-day expedition, during which an unsupported crossing was made from the west coast to the east coast of Greenland. The first person to cross Greenland was the Norwegian, Fridtjof Nansen, in 1888 with five other companions. Since then, many expeditions have been completed on skis, with participants hauling the necessary equipment on “human-powered” sledges. Although the crossing is not particularly demanding from a technical point of view, it does require self-reliance for about a month, as there are no human settlements along the route. Potential dangers include blizzards, often very low temperatures, crevasses, and polar bears near the —the largest carnivores on earth—and physical fatigue.

The expedition started near Kangerlussuaq on the west coast, ending at a fjord near the village of Isortok on the east coast: nearly 560 km of actual distance travelled (although as the crow flies the distance is shorter). Within the context of this expedition, a scientific project called GREENLAND EXPEDITION LAB 2019 was implemented. Our tested hypothesis was to demonstrate that there is a positive correlation between PC and vagal cardiac response, through the analysis of two HRV indicators in the time domain: root mean square of successive (RMSSD) and standard deviation of all NN intervals (SDNN); and that therefore a high PC represents a favourable factor for recovery and stress management.

## Methods

### Participants

The experiment included four male participants of different nationalities (Italian, English, and French).

The number is very limited but it is explained by the rarity of these expeditions and therefore by the low number of subjects involved in general in similar activities. Often then these factors are joined by unforeseen events that affect the implementation of the measures themselves. In the specific case, there were seven participants in the crossing: the doctor, the only woman on the expedition, did not participate in the study in order not to include gender variables.

The data of two other subjects were discarded as incomplete due to malfunctions of the heart rate monitors related to the cold and the difficulty of constantly recharging them for a total period of almost a month.

The age of the four remaining subjects ranged from 33 to 58 years and their weight at the time of departure ranged from 72 to 106 kg (see Table 1). All subjects were expertly trained and highly experienced in expeditions. The oldest subject had reached the North Pole on an English expedition ten years earlier while the youngest was the record holder for crossing the Atlantic Ocean in a rowing boat from the Canary Islands to the Caribbean in the annual “Talisker Whisky Atlantic Challenge.”

**TABLE 1:** Subjects’ characteristics.

	Age	Height	Years of adventure experience	Weight (kg) at departure from Kangerlussuaq	Weight (kg) on arrival at Isortok
Subject 1	33	192	10	106.4	99.1
Subject 2	35	185	5	85.5	78.9
Subject 3	56	174	10	72.4	69.4
Subject 4	58	180	10	92.8	86.1

### Route and Walking Data

The explorers used laminated cross-country skis with heavy boots (Alfa brand, Norway) attached and each subject pulled their own sledge with all the equipment necessary for the crossing.

Progression was monitored using data obtained from the Global Positioning System (GPS). The GPS used were two: a Garmin Gpsmap 64 ST and a Garmin InReach Mini that can also be used for emergency satellite signals. In case of discrepancy in the measurements, an average was made between the two daily measurements. The number of hours spent walking per day—including short break times and lunch breaks—varied between 4 h (when walking was interrupted by blizzards) and 14 h. Daily distance travelled varied from 3.73 km (on the initial day of crossing the Ice Fall in order to climb the canopy) to 40.29 km. At the outset, the weight of each sledge was almost exactly the same (irrespective of each participant’s body mass), about 65 kg (see Table 2). This convergence in terms of weight was due to the fact that participants had standard single daily rations. Moreover, they had almost exactly the same minimum personal equipment. Shared equipment (one tent for two persons) as well as fuel, stoves, and pots were equally distributed. On arrival at Isortok, the sledges weighed approximately 39 kg. The average hourly speed during the day was always under 3 km/h with only two exceptions during the last few days (thanks to lighter sledges and slightly downhill slopes). The type of snow affected the speed of progression; in particular, fresh snow made the effort much greater. A few times, winds exceeded 20 m/s (72 km/h); under these circumstances, it was deemed appropriate to stop and set up camp.

**TABLE 2:** Daily characteristics of the expedition.

Dates	Day	km	Hours of walking in the day (*)	Speed	Slope %	Pulka Weight (estimate) (kg)
01 May	1	6.3	7	1.30	1.60	65
02 May	2	3.73	7	0.77	3.50	64
03 May	3	9.02	9	1.39	1.50	63
04 May	4	17	8	3.00	1.30	62
05 May	5	7.77	4	3.33	1.10	61
06 May	6	16.88	8	2.98	0.90	60
07 May	7	20.3	9	3.12	0.90	59
08 May	8	25.45	11	3.12	0.40	58
09 May	9	22.28	11	2.73	0.60	57
10 May	10	22.54	11	2.76	0.70	56
11 May	11	30.15	15	2.62	0.50	55
12 May	12	10.7	5	3.38	0.00	54
13 May	13	11	5	3.47	–0.10	53
14 May	14	11.63	6	2.91	–0.20	52
15 May	15	19.42	9	2.99	0.40	51
16 May	16	32.46	14	3.04	0.40	50
17 May	17	24.94	10	3.40	0.20	49
18 May	18	28.93	11	3.54	0.10	48
19 May	19	28.04	12	3.12	–0.30	47
20 May	20	18.03	11	2.21	–0.60	46
21 May	21	19.44	9	2.99	–0.20	45
22 May	22	13	8	2.29	–1.00	44
23 May	23	21.55	11	2.64	–0.40	43
24 May	24	29.59	10	4.04	–0.60	42
25 May	25	40.29	14	3.78	–0.80	41
26 May	26	37.83	13	3.85	–0.80	40
27 May	27	32.65	9.5	4.72	–3.50	39
	Tot.	560.92	257.5	2.94	0.21	

*Including standard break and lunch time.

### Measurements and Instruments

#### Perceived Control

Perceived control was measured using the Pearlin Mastery Scale (Pearlin and Schooler, 1978), which measures the degree to which an individual views his or her life events and circumstances as being under his or her personal control (mastery) rather than fatalistically governed. The scale comprises 7 items (e.g., “There is really no way I can solve some of the problems I have”; “I have little control over the things that happen to me”; “What happens to me in the future mostly depends on me”), with response options distributed along a 7-point Likert scale.

The participants completed the scale every other day, at the end of the day, once they had stopped and set up camp with tents for the night. Additionally, beat-by-beat heart rate measurement was performed every other day on waking up, i.e., the following morning. PC was influenced by daily events, including the difficulty of the walk, the presence of wind or other threats (e.g., crevasses or polar bears), state of health (blisters, pain, backache, frostbite), and fatigue. All these variables had an impact on the participants’ perception of likelihood of success, that is whether they would be able (or not) to reach the coast in time, before food supplies and fuel (to melt water) ran out. If not, they would have been forced to call in a rescue expedition.

#### Other Subjective Measures

Together with PC, a self-reported perception of the effort exerted during the daily trek was also asked to the subjects by means of the Borg scale (CR 100) (Borg and Borg, 2002). In addition, each participant was asked to specify on a special sheet the most significant event of the day, whether in a positive or negative sense (see Supplementary Material).

#### Heart Rate Variability

Beat-by-beat heart rate was measured every other day on waking up, i.e., the morning after completion of the subjective scales. Each subject was equipped with a Garmin Forerunner 920 XT heart rate monitor (Cassirame et al., 2017). The recording was carried out by the participants themselves while still lying in their sleeping bags (tents were narrow and icy), having worn the HR wristband the whole night. The duration of the recorded time series was 8 min. After completion of the expedition, all time-series were transfer to a laptop to perform HRV analyses using KUBIOS HRV Premium software.

The beat-by-beat HR time series data were analysed considering both frequency and time domains. In particular, we considered two time-domain indexes, RMSSD and SDNN. RMSSD is the square root of the sum of squared differences of sequential NN pairs (normal RR intervals). It is an index of parasympathetic activity within autonomic regulation: the higher the RMSSD value, the more active the parasympathetic regulation (Baevsky and Chernikova, 2017). RMSSD is considered more reliable than SDNN as an index of measurement of short-term variation in HRV (Malik et al., 1996). SDNN provides the standard deviation of normal interbeat intervals measured in milliseconds and reflects the influence of all factors contributing to HRV (it is therefore correlated with total power in the frequency domain) (Malik et al., 1996). In Buchheit’s review of methods for monitoring athletic recovery, RMSSD is reported as one of the most reliable indices of recovery, highlighting its low influence on current respiratory rates (Buchheit, 2014). Numerous other researches come to the same conclusions (Plews et al., 2013; Esco et al., 2018).

We also considered four indexes in the frequency domain: LF, HF, VLF, and TP. TP is a sum of power in the HF, LF, and VLF ranges.

## Statistical Analyses

Due to our small sample size and to the repeated measures characteristics of the protocol, we conducted repeated measures correlations (Bakdash and Marusich, 2017) between PC, BORG scale and HRV markers. The data were processed using DAY 1 as a reference, assessing intra-individual variation on subsequent days. Repeated measures correlation is a statistical technique for determining the common within-individual association for paired measures assessed on two or more occasions for multiple individuals (Bakdash and Marusich, 2017). This technique can handle repeated measures’ data without violating independence assumptions or requiring the averaging of the data first. It allows for assessing a common association across individuals, specifically a homogenous intra-individual linear-association relationship between paired measures. Like a Pearson correlation coefficient (r), the rmcorr coefficient (rrm) is bounded by -1 to 1 and represents the strength of the linear association between two variables (the null hypothesis for rmcorr is ρrm = 0, and the research/alternative hypothesis is ρrm ≠ 0) (Bakdash and Marusich, 2017). Confidence intervals (CI) were computed by using the optional parameter proposed by the rmcorr package. Complementary, rmcorr takes advantage of multiple data points per participant, leading to have greater statistical power than a standard Pearson (or Spearman) correlation. This point is critical for our purpose, because low power typically overestimates effect sizes (e.g., Button et al., 2013). More precisely, Power for rmcorr increases exponentially when either the value of k (the number of repeated observations) or the value of N (the total number of unique participants) increases (Bakdash and Marusich, 2017). Based on our protocol and on the rmcorr strength, the repetition of measures helps to compensate our small sample size and allow (without generalisation) to make some statistics in order to better understand the links that exists between PC and HRV markers. Finally, the following criteria were adopted to interpret the magnitude of the correlation between the test measures: <0.1, trivial; 0.1–0.3, small; 0.3–0.5, moderate; 0.5–0.7, large; 0.7–0.9, very large; and 0.9–1.0, almost perfect (Hopkins et al., 2009). An alpha level of 5% was used in all the analyses.

## Results

**FIGURE 1:**
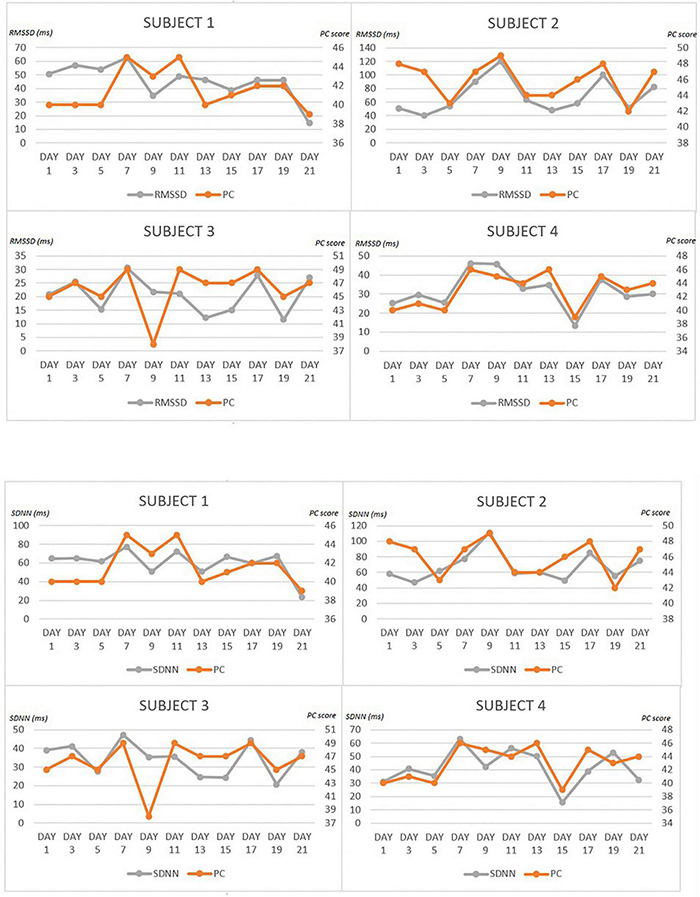
Trend ot the PC values in relation to SDNN and RMSSD for each individual subject.

Table 3 shows the Repeated measures correlation values between PC and time-domain and spectral indexes. Graphs were then inserted which represent, for each individual subject, the trend of the PC values in relation to the two main indices examined in the time domain: SDNN and RMSSD (Figure 1). The tables with values of the perceived effort, Mean RR, SDNN, RMSSD, and PC on all even days, are given in Supplementary Material.

**TABLE 3:** Repeated measures correlation values between PC and time-domain and spectral indexes.

Non-specific dimension				
***Perceived control***				

	**rrm**	**df**	**p**	**Ci**
**RPE**	–0.16	39	0.33	–0.45; 0.17
**MeanRR**	0.16	39	0.31	–0.16; 0.45
**SDNN**	0.51	39	0.001	0.22; 0.71
**RMSSD**	0.46	39	0.002	0.16; 0.67
**TINN**	0.44	39	0.004	0.14; 0.66
**TPms**	0.31	39	0.049	0.00; 0.57
**LogVLF**	0.35	37	0.025	0.04; 0.61
**LogLF**	0.39	37	0.015	0.07; 0.63
**LogHF**	0.35	37	0.03	0.02; 0.60
**LogTP**	0.35	37	0.027	0.03; 0.61
**DLpreHRV**	0.33	39	0.034	0.02; 0.59

*RPE, rate of perceived exertion; MeanRR, average R-R interval duration in a measurement, SDNN, standard deviation of all NN intervals; RMSSD, root mean square of successive; RR, intervals differences; TINN, baseline width of the RR intervals histogram; TP, total spectrum power; VLF, very low frequency power; LF, low frequency power, HF, high frequency power, DLpreHRV, daily load i.e., mechanical work of the Pulka expressed in Kilojoules during the day before the HRV measurement.*

## Discussion

The tested hypothesis in this study was that during a 27-day expedition across Greenland, a positive correlation between PC and vagal cardiac response exists and that therefore a high PC represents a favourable factor for recovery and stress management. The results seem to confirm the hypothesis as strong positive correlations have been observed between PC and vagal indexes (RMSSD, SDNN).

Our results also shown that at an intraindividual level links exists between the secondary cognitive appraisal (i.e., perceived control) and vagal indexes. Based on the Transactional Model of Stress and Coping (Lazarus and Folkman, 1984), individual’s appraisals of a situation is a core psychological process that explains within-person variations in adaptation and performance. Perceived control has been shown as a predictor of athletes’ stress and recovery states when the general aim is to be ready to compete and hence to give an all-out effort (Vacher et al., 2019). Complementary, a recent study shown that the linear trajectory of parasympathetic markers varying according to levels of PC (Vacher et al., 2018). Based on this literature, one’s can argue the individual’s cognitive appraisal of the situation can lead to physiological modifications, more or less suitable for performance. This means that the more people perceive themselves, their surroundings and their goals as controllable (and therefore the less they feel threatened), the less the activation of the hypothalamus-pituitary-adrenal (HPA) axis will be (Dallman, 1993; Stults-Kolehmainen et al., 2014). From what has just been said, we can logically deduce that the PC is influenced by the experience of subject and his fitness level.

Examples of events that caused a drop in PC and vagal indexes were observed in the notes in which the participants recorded emotional events of the day (see Supplementary Material): “Another day where we stayed in the tent in the morning because of the wind. We moved just after lunch and only took 5/6 steps. At the camp I nearly burned the tent with the stove and had to get help from 1r. My nose has a very bad mark on it, maybe it is frostbite” (Subject 2, Day 7). Or: “Sledge in the crevasse. It seems to me to be slower and clumsier than all the others” (Subject 3, Day 2). Or: “First time I had to leave the ski line to rest—disappointed in myself!” (Subject 4, Day 8). In the same notes it was also possible to detect events or experiences which were, on the contrary, presumably ascribable to an increase in PC: “The victorious kilometres made in the storm!” (Subject 3, Day 7); or: “Feeling of going fast without fatigue” (Subject 3, Day 8).

However, no positive correlation was found between PC and perceived effort as measured by the Borg CR-100 scale. In a way, PC “models” the different emotional meaning that is attributed to effort. In the reports of the significant events of the day, there were examples of situations where a high perceived effort was experienced with a positive sense of high control over one’s own performance and over the progression of the crossing: “Been able to do 30 km for the first time (in 15 h)” (Subject 3, Day 6, in relation to a perceived effort of 65). Or: “Big day—40 km covered. My birthday” (Subject 1, Day 8, perceived effort = 70). In other cases, a high feeling of exertion is concomitant with a decrease in PC: “First time I had to leave the ski line to rest—disappointed in myself!” (Subject 4, Day 8, perceived effort = 70).

It is conceivable, and could be the subject of future studies, that HRV, PC, and perceived effort show a varied trend because:

1. this specific study relies on a real expedition, and hence it is not a race (Fazackerley et al., 2019). The purpose if thus completely different (in the first case the aim is to safely finish with the teammates, while in the second the aim is to beat an opponent with most of the time an individual or collective all-out effort). Hence, we can expect that the participants use various strategies to keep their resources in balance.
2. the duration of the crossing itself is such that the subjects must maintain a strenuous balance between walking speed, duration of energy resources and time to reach the final goal.

Due to the small number of subjects these results should be considered as a starting point for a more in-depth study analysing the relationship between HRV-PC and performance. Once the positive correlation between CP and cardiac vagal regulation is confirmed, we should carry out specific training interventions before such expeditions with the aim of improving the coping skills and resilience of the athletes involved.

This study suggests that in addition to the usual athletic training programmes, there could be a need for specific training that focuses on the cognitive and emotional skills of subjects (Trabucchi, 2005).

## Conclusion

The existence of a positive correlation between PC and cardiac vagal regulation is of great interest to individuals immerged into extreme situations, because it can affect performance or prevents maladaptive states or injuries. Since the cognitive appraisal is sensitive to experience and can be modified through specialised learning and training, it is convenient for members of extreme expeditions to adopt these forms of preparation in a systematic way, alongside the normal athletic or technical training protocols. Further research could focus on the implementation of mental preparation protocols for expedition teams and verify their effectiveness.

## Data Availability Statement

The original contributions presented in the study are included in the article/Supplementary Material, further inquiries can be directed to the corresponding author.

## Ethics Statement

The studies involving human participants were reviewed and approved by Comitato di Ateneo per la ricerca sulla persona (CARP, Univesity of Verona). Written informed consent for participation was not required for this study in accordance with the national legislation and the institutional requirements. Written informed consent was not obtained from the individual(s) for the publication of any potentially identifiable images or data included in this article.

## Author Contributions

PT had the idea of the research and carried out the measurements and wrote the manuscript. AS, LM, and PV designed the study. LM, PV, and BP performed the numerical and statistical calculations. FS supervised the project. All authors discussed the results and contributed to the final manuscript.

## Conflict of Interest

The handling editor declared a shared affiliation with several of the authors PT, AS, BP, and FS at time of review. The remaining authors declare that the research was conducted in the absence of any commercial or financial relationships that could be construed as a potential conflict of interest.

## Publisher’s Note

All claims expressed in this article are solely those of the authors and do not necessarily represent those of their affiliated organizations, or those of the publisher, the editors and the reviewers. Any product that may be evaluated in this article, or claim that may be made by its manufacturer, is not guaranteed or endorsed by the publisher.
