# Supplementary material for: Relationship Between Cognitive Appraisal of Control and Cardiac Vagal Regulation During an Unsupported Ski Crossing of Greenland
Source: Front Physiol. 2022 Apr 8;13:804710. doi: 10.3389/fphys.2022.804710 (PMC9024293; doi:10.3389/fphys.2022.804710)
Supplement: Supplementary file 1 [file Data_Sheet_1.PDF]

SUPPLEMENTARY MATERIAL 1- DAILY SHEETS WITH EMOTIONAL EVENT OF THE DAY

Subjects		BORG	EMOTIONAL EVENT OF THE DAY before HRV day
1	DAY 1	30	Wet Feet in the icefall
	DAY 3	35	Felt Hungry
	DAY 5	30	running after tent pole bag and storm in the morning
	DAY 7	35	Very Hot
	DAY 9	35	Easy day
	DAY 11	50	Big day. 12 legs, arrived at Dye-2
	DAY 13	25	Easy day - only did 6 shifts
	DAY 15	70	Big day - 40 km covered. My birthday ;)
	DAY 17	40	Moderate effort
	DAY 19	40	Moderate effort
	DAY 21	50	Strong effort
2	DAY 1	45	My ankle hurted a lot in the evening (I think mostly due to the crampons and icy patches), but it was a good day and I didn't feel tired. I am not worried for the future as when I walk the ankle is good.
	DAY 3	35	We directly started on the skies, which was great. In the camp, it is the first day I washed myself (feet and bottom) which felt great.
	DAY 5	45	Not a very good day for me. I had pain in my big toe nail as it was compressed in the shoe. I don't feel tired though.
	DAY 7	60	My toe is better, which is good. The day was really warm and I find it harder than on the previous cold and windy days since we sweat a lot and got dehydrated faster.
	DAY 9	50	It was a good day as we were in the white. It gave a really good feeling of being in another world. I prefer also that it is not too warm.
	DAY 11	65	We set-off early (around7) to reach DYE2. There were a lot of wind. We struggled to arrive at the radar station
	DAY 13	40	Another day where we stay in the tent in the morning because of the wind. We moved just after lunch for 5/6 legs only. At the camp I nearly burned the tent with the stove and had to get help from 1r. My nose has a very bad mark on it, maybe it is frostbite.
	DAY 15	40	The morning was good, sunny and too warm but as the day went on it started to be very warm (and therefore sweaty). We struggled with the last two legs but we managed. During the day there were some birds flying over the glacier. It was nice since it was the first animal I saw since the raven in the icefall.
	DAY 17	50	The snow has been falling and it is harder to move.
	DAY 19	55	There were a lot (too much) of powder snow. It was difficult at the front. I injured my ankle at the end of the afternoon, wich impacted my moral. I felt like we have to do more to cover the different injuries, equipment failure, tiredness and there it put more pressure on those which are still valid (but for how long?) - 55% because the day was not hard at all but still strong because of my ankle.
	DAY 21	55	Still too much snow. I feel a bit better on my ankle although I still think it might be very hard to finish the expedition with Pietro, Simon tired, 1r shoes broken and my ankle in a bad shape.
3	DAY 1	50	pools of water in the glacier
	DAY 3	50	sled in the crevasse. It seems to me to be slower and more clumsy than all the others
	DAY 5	55	very strong wind!!
	DAY 7	30	lead the group with the compass
	DAY 9	60	not being able to prepare everything in time and feel the pressure of the group to hurry
	DAY 11	65	be able to do 30 km for the first time (in 15 hours)
	DAY 13	55	The victorious kilometers made in the storm!
	DAY 15	30	feeling of going fast without fatigue
	DAY 17	30	being able to fairly finish my legs
	DAY 19	55	the descent begins to appear!
	DAY 21	45	to have recovered some of my energy. Kristin's strength beating the track
4	DAY 1	60	Getting feet and pulk contents very wet in the icefall.
	DAY 3	50	Knee swollen after fall on ice and swollen/painful; stress of knowing if this will cause me a problem
	DAY 5	60	Very cold with headwind. Stupid mistake losing glove; minor frostnip
	DAY 7	60	Very hot and got a little dehydrated in heat!
	DAY 9	55	Developed chaffing on leg from heat/sweat. Worried it may cause a problem for me!
	DAY 11	55	Very cold wind in face. Worried about lack of food/poisoning!
	DAY 13	60	Heptane poisoning of food confirmed. Angry and disappointed nothing done about this so far!
	DAY 15	70	First time I had to leave the ski line to rest - disappointed in myself!
	DAY 17	65	Had some kit taken from me by Sebastian - angry at way it was done. Diarrhea (from food poisoning?)
	DAY 19	85	No sleep at all - no idea why!
	DAY 21	80	At the end of my abilities and energy (especially when roped up).

SUPPLEMENTARY MATERIAL 2- DATA

**Mean RR**					**SDNN**				**RMSSD**			
**DAY**	***S1***	***S2***	***S3***	***S4***	***S1***	***S2***	***S3***	***S4***	***S1***	***S2***	***S3***	***S4***
Basal	956.0	1022.1	1167.0	1181.0	46.,7	64,.6	36,.2	29,.2	39.2	61.0	19.3	24.0
DAY 2	932.0	916.8	1145.0	1098.0	64,.6	58.0	39.0	31,.1	50.6	50.9	20.8	25.1
DAY 4	997.0	775.8	1169.0	1077.0	65.0	47,.1	41,.1	40,.8	57.0	40.3	25.5	29.6
DAY 6	993.0	893.0	1094.0	1065.0	61,.8	61,.5	27,.5	35,.5	54.0	54.2	15.3	25.5
DAY 8	992.0	1004.0	1213.0	1103.0	77,.2	77,.4	47,.3	63,.2	62.5	90.3	30.6	46.0
DAY 10	928.0	1044.0	1140.0	1010.0	50,.7	111,.4	35,.4	42,.3	34.7	120.3	21.7	45.7
DAY 12	839.0	907.0	1028.0	1107.0	72,.5	58,.5	35,.6	56,.2	49.0	63.3	21.1	32.8
DAY 14	991.0	879.0	1052.0	1125.0	50,.6	59,.7	24,.6	50,.1	46.3	48.0	12.2	34.7
DAY 16	920.0	849.0	1066.0	1037.0	66,.5	50.0	24,.4	15,.5	38.7	58.0	15.1	13.4
DAY 18	1018.0	1098.0	1154.0	1103.0	60.0	85,.1	44,.3	38,.8	46.2	100.2	27.8	37.5
DAY 20	954.0	993.0	1000.0	1081.0	67,.4	55,.3	20,.7	53.0	46.1	51.9	11.6	28.7
DAY 22	742.0	998.0	1149.0	1167.0	23,.2	75.0	38,.1	32,.3	14.6	82.7	27.0	30.0
Final	1067.0	1156.0	1161.0	1119.0	43.0	62,.2	27,.2	43,.9	51.8	66.9	18.9	43.0
AVG.	***948.3***	***964.2***	***1118.3***	***1097.9***	***57,.63***	***66,.6***	***33,.99***	***40,.91***	***45.4***	***68.3***	***20.5***	***32.0***
ST. DEV.	***78.5***	***92.8***	***65.3***	***48.3***	***14,.42***	***17,.99***	***8,.426***	***13,.31***	***12.3***	***24.41***	***6.2***	***9.2***

	**DAY**	***S1***	***S2***	***S3***	***S4***		**DAY**	***S1***	***S2***	***S3***	***S4***
**BORG CR-100**	DAY 1	30	45	50	60	**PERCEIVED CONTROL**	DAY 1	40	48	45	40
	DAY 3	35	35	50	50		DAY 3	40	47	47	41
	DAY 5	30	45	55	60		DAY 5	40	43	45	40
	DAY 7	35	60	30	60		DAY 7	45	47	49	46
	DAY 9	35	50	60	55		DAY 9	43	49	38	45
	DAY 11	50	65	65	55		DAY 11	45	44	49	44
	DAY 13	25	40	55	60		DAY 13	40	44	47	46
	DAY 15	70	40	30	70		DAY 15	41	46	47	39
	DAY 17	40	50	30	65		DAY 17	42	48	49	45
	DAY 19	40	55	55	85		DAY 19	42	42	45	43
	DAY 21	50	55	45	80		DAY 21	39	47	47	44
